# The Impact of an Educational Video on Clinical Trial Enrollment and Knowledge in Ethnic Minorities: A Randomized Control Trial

**DOI:** 10.3389/fpubh.2019.00104

**Published:** 2019-04-26

**Authors:** Jeannine S. Skinner, Alecia M. Fair, Alexis S. Holman, Alaina P. Boyer, Consuelo H. Wilkins

**Affiliations:** ^1^Interdisciplinary Gerontology Program, Department of Psychological Sciences, University of North Carolina at Charlotte, Charlotte, NC, United States; ^2^Meharry-Vanderbilt Alliance, Vanderbilt University Medical Center, Nashville, TN, United States; ^3^Sarah Cannon Research Institute, Sarah Cannon, Nashville, TN, United States; ^4^National Health Care for the Homeless Council, Nashville, TN, United States; ^5^Department of Medicine, Meharry Medical College, Nashville, TN, United States

**Keywords:** clinical trials, ethnic groups, minority groups, patients, cancer, education, multimedia

## Abstract

**Introduction:** Innovative methods to increase awareness about clinical trials and address barriers associated with low participation among racial/ethnic minorities are desperately needed. African Americans comprise 5% of all clinical trial participants, and Hispanics make up 1%. Use of multimedia educational material has shown promise as an effective strategy to increase minority clinical trial enrollment. However, this approach has not been broadly implemented. We tested the effect of a video educational program on clinical trial knowledge and enrollment in a sample of oncology outpatients.

**Methods:** A randomized controlled trial was conducted with 63 oncology patients without previous history of clinical trial participation. Participants were randomly assigned to the intervention, to watch a clinical trial educational video in the office, or to the control group which did not receive in-office education. The Clinical Trial Knowledge survey was administered before the intervention and 1 week after the intervention. Participation in clinical trials was assessed 1-year post study participation. Results for white participants and ethnic minorities were compared. Ethnicity was self-reported through the electronic health record and confirmed by self-reporting on questionnaire.

**Results:** Sixty-three participants were recruited in this study. At 1-year follow-up, 3 participants enrolled in clinical trials in the study group which had received office-based video intervention and 2 participants enrolled in the control group (*Z* = 0.39, *p* = 0.69). These results were not statistically significant. Impact of the intervention by ethnicity could not be assessed due to low total clinical trial enrollment. The video intervention did not change knowledge, attitudes, or barriers as measured by the Clinical Trial Knowledge Survey. Minority participants did report significantly more negative beliefs and barriers to participation than white participants.

**Conclusions:** Increasing awareness and knowledge about clinical trials in underrepresented communities is an important step to providing opportunities for participation. Future studies should focus on how to address the negative expectations of clinical trials and the greater information needs in minority populations. Tailored or personalized messaging may address negative perceptions of clinical trial participation.

## Introduction

Clinical trial participation is low especially among racial/ethnic minorities. Studies show African Americans comprise 5% of all clinical trial participants, and Hispanics make up 1% (1). Only 3–5% of cancer patients enroll in clinical trials, with racial/ethnic minorities making up a small fraction of enrollees (2, 3). Cancer incidence rates are typically lower among racial/ethnic minorities than non-Hispanic Whites, yet, minorities have a higher risk of mortality and shorter survival than non-Hispanic Whites (4, 5). For this reason, minority participation in clinical trials has important implications for improving health equity and addressing ethnoracial health disparities (6).

Limited awareness and knowledge about clinical trials (7, 8) are key personal factors that impede a prospective participant's ability to decide on whether he/she would like to participate in a clinical trial (8). Studies show clinical trial awareness and knowledge is associated with sociodemographic and economic factors, such that younger individuals, Whites and persons of higher socioeconomic status (9) have greater clinical trial awareness and knowledge, and are more likely to participate in clinical trial than persons who do not fit this sociodemographic profile. As such, initiatives focused on clinical trial education (10) and increasing health and scientific literacy among minorities (11) may be particularly effective in reducing barriers to clinical trial participation in these groups. Video-based education may be especially effective in increasing knowledge about research (12), improving attitudes toward research (13), and increasing enrollment in research studies (1) among hard-to-reach populations. Clinical research education provided through multimedia may also be favored among those with limited health and scientific literacy (14, 15).

We undertook a study to (a) describe the utility of a clinical trial educational video in a diverse oncology patient population, and (b) examine the preliminary effect of an office-based clinical trial education video intervention on clinical trial knowledge, perceived barriers, and clinical trial enrollment. We also explored differences in clinical trial knowledge and barriers to participants between minority participants and White participants. The feasibility of implementing this intervention in a clinic setting was conducted using the Stages of Implementation Completion (SIC) measure (16). Findings from this study will add to the existing literature on effective strategies to increase minority group participation in clinical trial research.

## Materials and Methods

### Study Design

A randomized control trial design was conducted to determine preliminary effects of a clinical trial education video on clinical knowledge, perceptions of barriers to participating in clinical studies, willingness to participate in clinical trials and clinical trial enrollment. The Vanderbilt University Medical Center Institutional Review Board approved this study.

### Participants and Setting

A power size calculation (power of 0.80, a 0.05 significance level) based on a previous study (17) with similar goals and methodology indicated a sample size of 40 (20 for each group) would provide confidence that the resulting effect size represents that which would be expected in a fully powered study. We sought to include in our sample a matched proportion of participants of racial/ethnic minorities. Inclusion criteria were a diagnosis of malignancy, age of 18 years or older, English proficiency, and no previous history of clinical trial participation. Prospective participants were recruited from urology, hematology and breast specialty clinics of Vanderbilt Ingram Cancer Center (VICC).

### Intervention

Participants were randomized to the intervention (in-office video viewing group) or control, (DVD to take home, usual care group) using the web-based program Research Randomizer (4.0). Both groups received a clinical trial educational booklet and a copy of the DVD video on cancer clinical trials. The booklet provided a definition of clinical trial, descriptions of different types of clinical trials, and potential benefits and risks associated with participating in clinical trials. The video depicted oncology patient advocates sharing personal stories of participation in clinical trials and interviews with oncologists discussing the importance of cancer clinical trials. The video was created by the Vanderbilt-Ingram Cancer Center Office of Patient and Community Education and is distributed as an educational resource to new oncology patients. Using a tablet computer and headphones, the intervention (video viewing) group viewed the video while in the clinic. The control group was provided a copy of the booklet and video and given no further instruction.

### Measures

**Figure 1:**
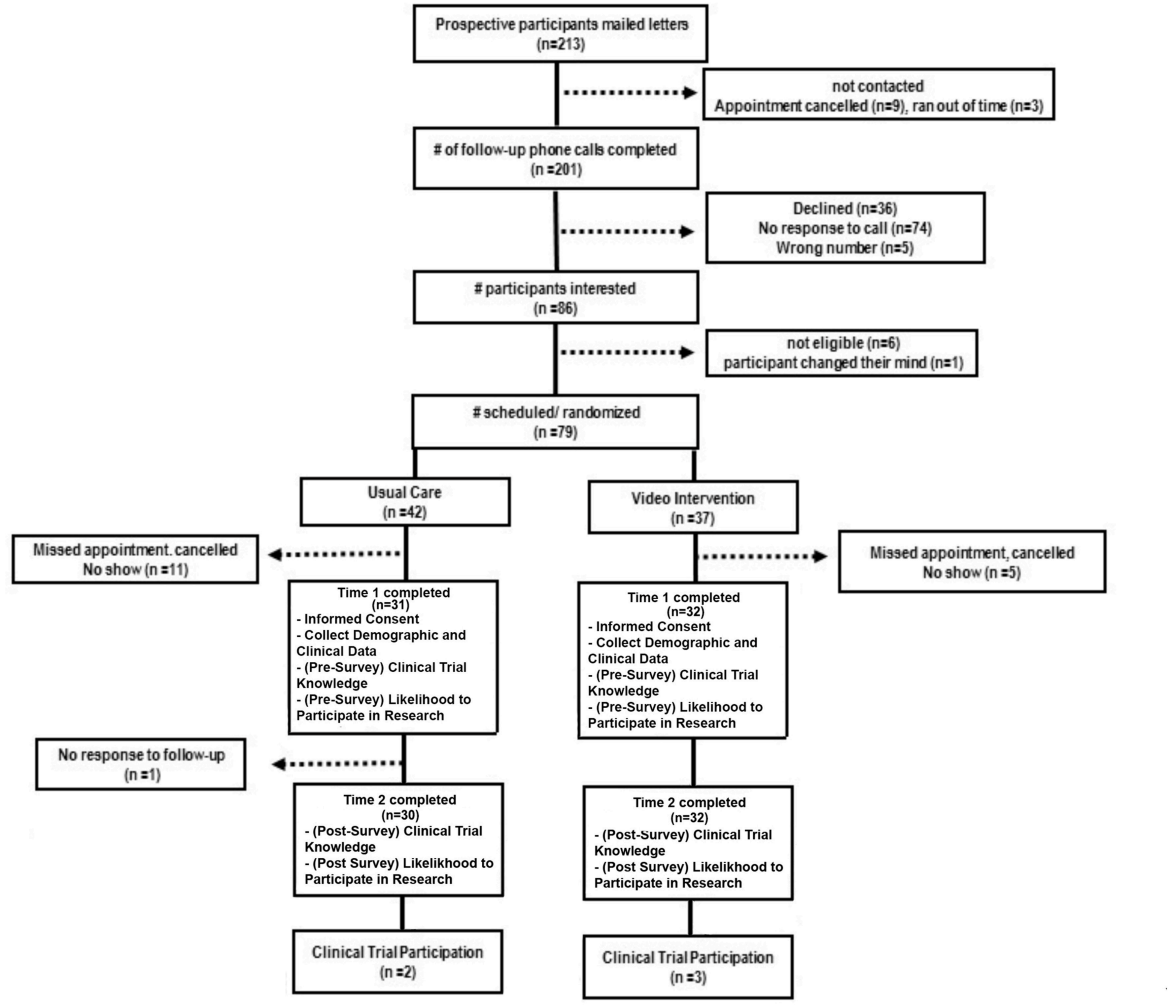
Participant flow.

After randomization, all participants were asked to complete a pre-survey prior to receiving the educational resource intervention. A post-survey was conducted by phone 1 week later. A participant flow diagram is provided in Figure 1. The 22-item Clinical Trial Knowledge survey was used to assess participants' awareness about clinical trial research (18). Survey items span 5 independent subscales [positive beliefs (4-items), safety (4-items), information needs (4-items), negative expectations (6-items), and patient involvement (4-items)] and are measured on a 5-point Likert scale ranging from 1 (*strongly disagree*) to 5 (*strongly agree)*. Perceived barriers to participation in clinical trials were assessed with 8-items on a 3-point scale ranging from 1 (*this is not a reason for me*), 2 (*this is a minor reason for me*), and 3 (*this is a major reason for me*) (unpublished *observations*, Patient-Centered Clinical Research Network, 2014). Participants were provided a list of reasons he/she may not want to participate in medical research studies and asked to indicate their feelings. Clinical trial knowledge and barriers to participation survey questions are provided in Supplementary Material. Enrollment in clinical trials was assessed 1-year post follow-up visit by review of VICC clinical trial participation electronic record. Study feasibility was measured using the Stages of Implementation Completion (SIC) measure an 8-stage, validated tool, measuring the implementation process across three phases (pre-implementation, implementation, and sustainability) (16).

### Procedures

Potential participants were identified using Subject Locator, a recruitment tool to identify potential research participants based on discrete study inclusion/exclusion criteria available in Vanderbilt University Medical Center clinical systems. Study participant recruitment was streamlined by pre-screening patients from a list of upcoming appointments at the Vanderbilt Ingram Cancer Center. The resulting subset of clinic patients matched our study criteria and followed recruitment work flow.

To ensure sufficient representation of racial/ethnic minorities, the prospective participant pool was oversampled for racial/ethnic minorities at a ratio of 2:1. Prospective participants received a recruitment letter by mail and a follow-up phone call to determine interests and confirm eligibility for study participation. Study appointments were scheduled before or after the patient's next clinic visit. Once study appointments were scheduled, staff randomly assigned participants to the intervention or control condition.

All participants completed the pre-survey using android-based tablet computers equipped with Talking Survey™ software. Talking Survey™^1^ is an integrated patient surveying and healthcare education system. Key features include multilingual abilities, voice-over question administration, voice response option, and audio-to-text transcription. Participant data was automatically transmitted to the secure Research Electronic Data Capture (REDCap) database (19). Voice-over survey administration and touch screen response were used. After completing the pre-survey, participants in the video-viewing group watched the 10-min clinical trial education video. Participants in the control group were provided with the educational booklet and a DVD copy of the video after completing the survey. One week after completing the pre-survey, all participants completed the post-survey via a phone call. Approximately 1-year after participating in the study, participant's VICC clinical trial record system was queried to determine whether the participant enrolled in a VICC-affiliated clinical trial study. Participants received $50.00 after completing the post-survey.

### Statistical Analyses

Results are reported using standard descriptive statistics. A mixed ANOVA was performed to assess the impact of the two interventions in the scores of the survey before and after the intervention. Race was converted into a dichotomous variable (whites/minorities) and included into the model as a covariate to assess its effects. Change scores from pre-survey to post-survey were calculated for each dependent variable and these values were used in the analyses. A series of one-way between-group analyses of variance were conducted to determine the impact of the experimental video vs. control on dependent variables. Between-group differences in clinical trial enrollment at 1-year post follow-up were analyzed using chi-square analysis. Significance was set at 0.05 level. Data was analyzed using Statistical Package for Social Sciences (version 23).

## Results

### Participants

**Figure 2:**
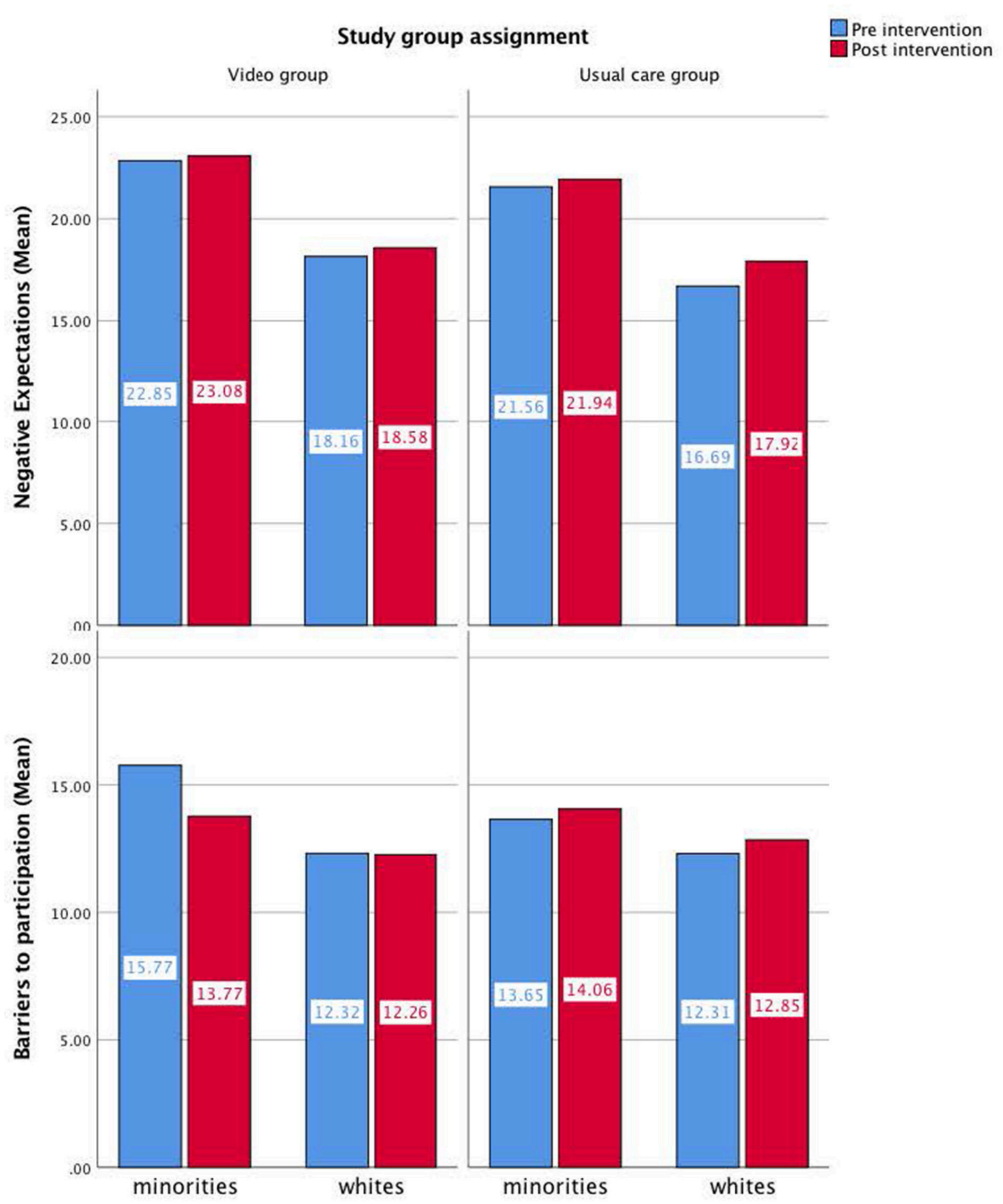
Pre and post intervention mean values of barriers to participation and negative expectations for each group of intervention by ethnicity.

Sixty-three participants were recruited. Baseline demographic characteristics are provided in Table 1. The intervention group and control group did not differ in age, gender makeup, level of education, or household income (*p* > 0.05). Hispanics were present in the video group but not the control group (*p* = 0.04). The majority of participants (53.2%) reported not reviewing either the educational booklet or video at home. There were no differences (*Z* = 0.39, *p* = 0.69) in the proportion of subjects who enrolled in a clinical trial after the video intervention (*n* = 3) and the control intervention (*n* = 2) (Table 2). This result could not be compared for minorities and whites due to low total clinical trial enrollment at the end of the study. We also studied the results of the survey by ethnic group to assess differences in clinical trial knowledge. Between groups analysis showed that the intervention did not have a significant impact on any of the domains assessed by the Clinical Trial Knowledge Survey or barriers to participation survey (Table 2). Within groups analysis did not demonstrate significant differences in baseline survey scores for the intervention and control groups. Within groups, minority participants were significantly more likely to harbor negative expectations of clinical trials (*F* = 23.21) and report higher barriers to participation (*F* = 7.97) irrespective of randomization arm (Figure 2 and Table 2).

**Table 1:** Baseline demographic characteristics for the total study population, intervention group, and control group.

**Demographics**	**Total** **(*n* = 63)**	**Intervention group** **(*n* = 31)**	**Control group** **(*n* = 32)**
Age (mean ± SD)	64.1± 10.4	65.8± 10.4	62.1± 10.3
Male % (*n*)	57.8 (37)	53.1 (17)	62.5 (20)
**Race % (*n*)**			
White	51.6 (33)	59.4 (19)	43.8 (14)
Black/AfAm	34.4 (22)	37.5 (12)	31.3 (10)
Asian	4.7 (3)	37.5 (12)	9.4 (3)
AI/AN	4.7 (3)	-	9.4 (3)
Other	4.7 (3)	3.1(1)	6.3 (2)
**Hispanic/latino % (*****n*****)**	7.8 (5)	15.6 (5)	0 (0)
**Vicc clinic % (*****n*****)**			
Urology	53.1 (34)	53.1 (17)	54.8 (17)
Hematology	21.9 (14)	18.8 (6)	25.8 (8)
Breast	23.4 (15)	28.1 (9)	19.4 (6)
**Education % (*****n*****)**			
Some high school	4.8 (3)	6.3 (2)	3.2 (1)
GED or high school	11.1 (7)	9.4 (3)	12.9 (4)
Some college	36.5 (23)	46.9 (15)	25.8 (8)
Associate degree	4.8 (3)	–	9.7 (3)
Bachelor's degree	15.9 (10)	15.6 (5)	16.1 (5)
Master's degree	19.0 (12)	18.8 (6)	19.4 (6)
Doctoral/professional degree	4.8 (3)	–	9.7 (3)
Other	3.2 (2)	3.1 (1)	3.2 (1)
**Reviewed educational resources at home % (*****n*****)**			
Read booklet	17.7 (1)	19.4 (6)	16.1 (5)
Viewed video	9.7 (6)	–	19.4 (6)
Read booklet + viewed video	19.4 (12)	22.6 (7)	16.1 (5)
Did not read booklet or view video	53.2 (33)	58.1 (18)	48.4 (31)
Lost to follow-up (*n*)	1	0	1

*p < 0.05; AfAm, African American; AI/AN, American Indian/Alaskan Native; NH/PI, Native Hawaiian/ Pacific Islander; VICC, Vanderbilt Ingram Cancer Center lost to follow-up= number of participants who did not complete post-survey.

**Table 2:** Pre- and post-survey results and mixed ANOVA analysis.

	**Intervention group**		**Control group**		**Total group**		**F within subjects**	**F between subjects**	
	**Pre**	**Post**	**Pre**	**Post**	**Pre**	**Post**	**Effect of intervention**	**Differences in baseline characteristics**	**Effect of ethnicity**
Positive beliefs	17.22(2.99)	17.06(2.26)	16.97(1.81)	17.23(2.31)	17.10(2.47)	17.15(2.27)	0.64	0.02	1.54
Safety	15.94(2.17)	15.84(2.11)	15.73(1.96)	15.80(2.12)	15.84(2.06)	15.82(2.10)	0.05	0.10	0.02
Information needs	16.63(3.45)	17.88(2.01)	18.00(1.95)	17.93(1.91)	17.29(2.89)	17.90(1.95)	3.46	1.28	3.63
Negative expectations	20.06(5.59)	20.41(4.14)	19.38(4.97)	20.14(4.46)	19.74(5.27)	20.28(4.26)	0.17	1.46	23.21^*^
Patient involvement	15.97(3.82)	15.84(2.08)	16.03(1.65)	16.17(1.74)	16.00(2.95)	16.00(1.92)	0.26	0.13	0.00
Barriers to participation survey	13.72(4.14)	12.88(3.06)	13.07(3.08)	13.53(3.01)	13.40(3.66)	13.19(3.03)	2.19	0.20	7.97^*^

*Results are reported as Mean(Standard Deviation) for five domains of Clinical Trial Knowledge Survey as well as the Barriers to Participation Survey*.

* *Statistically significant at p < 0.05*.

## Discussion

In this study we describe the utility of multimedia clinical trial educational resources and investigate the preliminary effect of a video educational program on clinical trial knowledge and subsequent enrollment in clinical trials in a diverse sample of oncology outpatients.

Our pilot randomized controlled trial revealed a null effect of video intervention on clinical trial knowledge or subsequent enrollment in clinical trials. In the control group, nearly half of participants reported not reading the educational booklet or watching the video provided to them, and only 19% in the video group reported reading the education booklet. These findings put into question the usefulness of multimedia technology as a method of health education and recruitment into clinical trials. Moreover, the study found that, within the randomization arms, minority participants were more likely to experience negative expectations of clinical trials and perceived more barriers to clinical trial participation than white participants which were not sufficiently addressed by the in-office video presentation. These findings align with prior work documenting minority group concerns about participation in clinical research, including clinical trials (1, 20–22).

These study findings suggest that alternative forms of communication be used to improve clinical trial knowledge and address barriers to participation which disproportionately affect minority groups (23). Some evidence suggests African Americans and Whites differ in their perceptions of effective communication channels for clinical trial information such that African Americans prefer to receive easy-to-understand clinical trial information through faith-based organizations and other in-person community-based channels, while Whites prefer to receive clinical trial information from doctors and print media (22). African Americans also express a preference for peer concordance representation in cancer information advertisements (24). Visual representation of extended family networks in cancer clinical trial educational videos provided to Hispanic cancer patients received greater clinical trial uptake, as it highlights cultural aspects of family input in patient decision-making central to Hispanic/Latino culture (20).

This study has several limitations. First, our sample size calculation based on previous studies

With similar goals and methodology indicated a sample size of 40, despite recruiting a total of 63 participants for this study. We did not account for stratification by ethnicity in our calculation of the sample size. This makes our results underpowered and hinders our ability to generalize findings, as studies with larger sample sizes are needed to confirm our results. Further studies should also apply stratified randomization and an adjusted sample size calculation to control for minority oversampling. Our results should be interpreted with caution as our study did not stratify randomization nor adjusted the sample size calculation by ethnicity.

To address the concerns about the combined analysis of the groups, we conducted between and within group analysis using ANOVA methods. Second, when assessing clinical trial enrollment 1-year post study participation, we did not query whether participants were asked to participate. It is possible some participants were never asked to participate in a clinical trial during the follow-up period. Despite these limitations, this study is unique in that it is one of the first to administer an interactive, tablet-based clinical trial educational video and survey in a clinic setting to a diverse patient population. Our use of interactive technology helps to circumvent research participation barriers related to lack of awareness about clinical trials, low literacy, and accessibility (25). Other strengths of our study include its RCT design and 50% minority group representation in the study population.

Interpersonal trust within the clinical relationship and medical establishment has been shown to be a significant factor in enhancing minority participation in clinical trials (26–28). A recent systematic review on barriers and facilitators to minority research participation recognized mistrust as a barrier to clinical trial participation (29). Despite expressions of mistrust, minority groups were willing to participate in clinical trials for altruistic reasons benefitting their family and community. Facilitators to clinical trial participation were illustrated as adapting culturally congruent practices such as addressing gaps in knowledge about research among a particular minority group (30, 31) translating study materials into appropriate languages and involvement of culturally and linguistically competent research staff (29, 32–35).

This study provides a guiding framework for future efforts to most effectively address and educate diverse patient populations about clinical trials for increased diversity in clinical research.

## Ethics Statement

This study was carried out in accordance with the recommendations of the Vanderbilt University Institutional Review Board, Behavioral Sciences Committee, a sub-committee of the Institutional Review Board with written consent from all subjects. All subjects gave written informed consent in accordance with the Declaration of Helsinki. The protocol was approved by the Vanderbilt University Institutional Review Board, Behavioral Sciences Committee, a sub-committee of the Institutional Review Board.

## Author Contributions

CW is the principal investigator of the study who designed the study and coordinated all aspects of the research including all steps of the manuscript preparation. She is responsible for the study concept, design, writing, reviewing, editing and approving the manuscript in its final form. JS and AH contributed in the study design and data collection. JS, AF, and AB contributed to the analysis and interpretation of data, drafting the work, writing the manuscript and reviewed and approved the manuscript. All authors read and approved the final manuscript.

### Conflict of Interest Statement

The authors declare that the research was conducted in the absence of any commercial or financial relationships that could be construed as a potential conflict of interest.

## Footnotes

^1^*Talking Survey LLC: Talking Survey*. Available online at: www.talkingsurvey.com.
