# Supplementary material for: The Impact of an Educational Video on Clinical Trial Enrollment and Knowledge in Ethnic Minorities: A Randomized Control Trial
Source: Front Public Health. 2019 Apr 26;7:104. doi: 10.3389/fpubh.2019.00104 (PMC6498183; doi:10.3389/fpubh.2019.00104)
Supplement: Supplementary file 1 [file Data_Sheet_1.docx]

**Supplementary Table.** Survey items for clinical trial knowledge survey and barriers to participation

|  | Clinical Trial Knowledge survey ( Jenkinson et al., 2005) |
| --- | --- |
| *Positive beliefs* | New and better treatments can only be produced if patients agree to take part in clinical trials. |
|  | Without the results from clinical trials, doctors would be less able to select the best treatment. |
|  | Pharmaceutical companies should ensure that valid clinical trials are conducted on every drug before it is generally available. |
|  | If most patients refused to take part in clinical trials, important developments in medicine would be seriously delayed. |
| *Safety* | Clinical trials are carried out according to strict rules to safeguard the interests of patients. |
|  | I assume that drug treatments that have been prescribed for me have already been thoroughly tested in clinical trials. |
|  | Clinical trials are only conducted on drugs for which there is already evidence to show that they are likely to be effective. |
|  | The conduct of all clinical trials is carefully regulated to ensure that the results are valid. |
| *Information Needs* | I would want as much written information as possible about clinical trials before I agreed to take part. |
|  | I would want to know before agreeing to take part that I would be free to withdraw from the clinical trial at any time. |
|  | I would want to know if I would be likely to get side effects by taking part in a clinical trial before I agreed to take part. |
|  | I would only take part in a clinical trial if I thought I understood everything about it. |
| *Negative Expectations* | I think I would find being in a clinical trial frightening. |
|  | I would only take part in the clinical trial if I thought my health would benefit. |
|  | I would only take part in a clinical trial if I thought I would not be inconvenienced by doing so. |
|  | I would only take part in a clinical trial if I knew which treatment I was going to receive. |
|  | I would only take part in a clinical trial if I was sure that the doctor treating me knew which treatment I was getting. |
|  | If I was satisfied with my current drug treatment, I would probably refuse to take a different drug in a clinical trial. |
| *Patient Involvement* | It is important for people like me to take part in clinical trials to confirm the value of new treatments and/or medical techniques. |
|  | I would take part in a clinical trial because the results should benefit patients like me in the future. |
|  | I think all patients who are eligible should be asked to take part in clinical trials. |
|  | Unless advised by their doctor to withdraw from a trial, all patients should co-operate fully until the trial is finished. |
| *Barriers to participation* | I do not want to be a "guinea pig." |
|  | The project was not recommended by my doctor. |
|  | My results may not be kept private or confidential. |
|  | The project will take a lot of my time. |
|  | I am not paid for taking part. |
|  | The project involves medical tests, like drawing blood or having x-rays. |
|  | The research might find out something bad about me. |
|  | There is no obvious benefit to me or my family. |

Clinical Trial Knowledge survey ( Jenkinson et al., 2005)
